# The Macrophage: A Disputed Fortress in the Battle against *Mycobacterium tuberculosis*

**DOI:** 10.3389/fmicb.2017.02284

**Published:** 2017-11-23

**Authors:** Christophe J. Queval, Roland Brosch, Roxane Simeone

**Affiliations:** Unit for Integrated Mycobacterial Pathogenomics, Institut Pasteur, Paris, France

**Keywords:** *Mycobacterium tuberculosis*, macrophages, phagosome maturation, cell death, cytosolic access

## Abstract

*Mycobacterium tuberculosis* (*Mtb*), the etiological agent of human tuberculosis (TB), has plagued humans for thousands of years. TB still remains a major public health problem in our era, causing more than 4,400 deaths worldwide every day and killing more people than HIV. After inhaling *Mtb*-contaminated aerosols, TB primo-infection starts in the terminal lung airways, where *Mtb* is taken up by alveolar macrophages. Although macrophages are known as professional killers for pathogens, *Mtb* has adopted remarkable strategies to circumvent host defenses, building suitable conditions to survive and proliferate. Within macrophages, *Mtb* initially resides inside phagosomes, where its survival mostly depends on its ability to take control of phagosomal processing, through inhibition of phagolysosome biogenesis and acidification processes, and by progressively getting access to the cytosol. Bacterial access to the cytosolic space is determinant for specific immune responses and cell death programs, both required for the replication and the dissemination of *Mtb*. Comprehension of the molecular events governing *Mtb* survival within macrophages is fundamental for the improvement of vaccine-based and therapeutic strategies in order to help the host to better defend itself in the battle against the fierce invader *Mtb*. In this mini-review, we discuss recent research exploring how *Mtb* conquers and transforms the macrophage into a strategic base for its survival and dissemination as well as the associated defense strategies mounted by host.

## Introduction

With over a billion deaths in the past 200 years, tuberculosis (TB) caused by *Mycobacterium tuberculosis* (*Mtb*) likely killed more people than any other infectious disease in the history of humanity (Paulson, 2013) and remains a major cause of death also in our era. *Mtb* is responsible for about 10 millions of new TB cases and 1.8 million deaths in 2015 (WHO, 2016). Estimations based on immunological tests suggest that about 2 billion people might be latently infected by *Mtb*. Statistically, 5–10% of latently infected individuals might then further develop active TB during their lifetime (Barry et al., 2009). TB primary infection occurs through inhalation of *Mtb*-containing aerosol droplets released by contagious individuals. After inhalation, *Mtb* rapidly reaches the lung’s alveolar space where it is preferentially taken up by alveolar macrophages (Armstrong and Hart, 1971; Warner and Mizrahi, 2007). Following macrophage phagocytosis, mycobacterial invaders deploy an army of factors, which circumvent macrophage defenses, to escape from macrophage killing and to replicate within these phagocytes (Ehrt and Schnappinger, 2009; Cambier et al., 2014). Manipulation of intracellular macrophage signaling also impacts the cytokine environment modifying the potency of protective immune response, setting up a *Mtb* tolerance by the host and favoring the intracellular survival of *Mtb* over time (BoseDasgupta and Pieters, 2014; Orme et al., 2015). Innate immune responses are essential for the outcome of TB infection as well as for the establishment of adaptive immunity (Torrado and Cooper, 2013; Orme et al., 2015). In lungs, the progressive establishment of an immune response during *Mtb* infection notably contributes to the aggregation of immune cells, forming an organized structure harboring macrophages at the center, surrounded by giant cells, T-lymphocytes, neutrophils, fibroblast, which is called a granuloma. A hallmark feature of the bacillus is its ability to remain concealed within host cells or/and within the granulomatous caseous necrotic centers, where it can persist during the long phase of TB latency (Barry et al., 2009). Establishment of an immune balance, orchestrated by both mycobacteria and host cells, is decisive for the outcome of the granuloma, which may either constrain the infection or promote its systemic dissemination (Ulrichs and Kaufmann, 2006; Davis and Ramakrishnan, 2009). The development of active pulmonary TB is tightly linked with a disordered immune balance, resulting in host’s inability to keep the infection under control (O’Garra et al., 2013).

The current TB drug regimen generally requires 2-months of treatment with four first-line drugs: isoniazid, rifampicin, ethambutol, and pyrazinamide followed by 4 months of treatment with isoniazid and rifampicin (WHO, 2016). However, the protracted nature of the TB treatment and an inappropriate patient compliance favor the selection of multidrug resistant strains (MDR-TB). The more recent emergence of extensively drug resistant strains (XDR-TB) represents, nowadays, a major threat (Van Rie and Enarson, 2006; WHO, 2016). Therefore, new and alternative means to control *Mtb* are urgently needed. A better understanding of the fundamental biology of this complex interaction of the bacterium and the host cell represents a challenge for the design of new strategies to improve control of TB.

This mini-review focuses on selected aspects of this host-pathogen interaction, which shows quite some resemblance to medieval battlegrounds, where fierce warriors tried to invade well-equipped fortresses with their weapons and ruses.

## Mycobacterial Artillery

*Mycobacterium tuberculosis* belongs to the phylum *Actinobacteria* and is coated by a unique cell envelope, which represents a remarkably impermeable and hydrophobic armor (Brennan and Nikaido, 1995) that is composed of a capsule, an outer membrane, also termed mycomembrane, a peptidoglycan layer, an arabinogalactan layer, and an inner plasma membrane. The mycomembrane consists of mycolic acids and selected extractible lipids, including phthiocerol dimycocerosates (abbreviated DIM or PDIM), diacyltrehalose (DAT), and polyacyltrehalose (PAT) (Chalut, 2016). Additionally, Mannosyl-phosphatidyl-*myo*-inositol-based glycolipids (PIM) and related lipoglycans such as lipomannan (LM) and lipoarabinomannan (LAM) are also abundantly present in the *Mtb* cell envelope as well as in the inner and outer membranes (Daffe et al., 2014). To ensure protein transport across this unusual cell envelope *Mtb* uses different secretion systems, some of which are also widely present in other bacteria, such as the general Sec systems and the Twin Arginine Translocation (TAT) pathway, whereas others are exclusively present in mycobacteria and in some distantly related versions in other species within the phyla *Actinobacteria* and *Firmicutes*. These latter systems are called ESX secretion systems (Gröschel et al., 2016) and are also known as Type VII secretion (T7S) systems (Abdallah et al., 2007). The *Mtb* genome carries 5 *esx* loci, which encode for 5 distinct systems (ESX-1-ESX-5). The molecular architecture of a representative ESX model (ESX-5 of *Mycobacterium xenopi*) was recently determined at 13Å resolution by electron microscopy (Beckham et al., 2017). This work showed four core proteins of the ESX-5 complex (EccB5, EccC5, EccD5, and EccE5), which assembled with equimolar stoichiometry into an oligomeric complex that displays sixfold symmetry (Beckham et al., 2017). Among the ESX systems, the *esx-1* locus is probably the most studied as it encodes the 6 kDa Early Secretory Antigenic Target (ESAT-6; EsxA) and the 10 kDa Culture Filtrate Protein (CFP-10; EsxB), considered as key virulence determinants of *Mtb* as well as strong T-cell antigens (Gröschel et al., 2016). Recent work has shown a concerted action of the ESX-1 secretion system of *Mtb* with DIM/PDIM in phagosomal rupture, leading to access of *Mtb* to the cytosol of the host macrophage (Augenstreich et al., 2017), a phenomenon recently discussed from different perspectives (Russell, 2016; Simeone et al., 2016) and described further below.

## Doorways of *Mtb* to Enter Into Macrophages

Interaction of *Mtb* with phagocytic cells mostly occurs through the recognition of Pathogen-Associated Molecular Patterns (PAMP) present at the bacterial surface by Pattern Recognition Receptors (PRRs) of the host cell such as Toll-Like Receptors (TLR), C-type Lectin Receptors (abbreviated as CLR or CTL), Fc Receptors (FcR), Scavenger Receptors (SR), and cytosolic DNA sensors (Satoh and Akira, 2016). Stimulation of PRRs leads to bacterial phagocytosis, the initiation of immune responses as well as the activation of numerous cellular processes such as apoptosis, antigen processing/presentation, inflammasome activation, phagosome maturation, and autophagy (Lugo-Villarino et al., 2011; Mortaz et al., 2015). The interaction between TLR and *Mtb* leads to phagocyte activation without immediate ingestion of mycobacteria. Recognition of specific mycobacterial structures, such as lipoproteins 19 kDa, LM, LAM, and PIM was reported to be established by TLR2 (Quesniaux et al., 2004), which is consistent with observations that TLR2-mediated recognition is diminished by the presence of Lipooligosaccharide (LOS) in *Mycobacterium canettii*, the smooth variant of tubercle bacilli (Boritsch et al., 2016). It is noteworthy that loss of LOS production during the evolution of tuberculosis-causing mycobacteria has resulted in the rough colony morphology of *Mtb* strains, which apparently contributed to stronger recognition of *Mtb* by TLR2 (Boritsch et al., 2016). Moreover, unmethylated CpG motifs in bacterial DNA were reported to be recognized by TLR9 (Bafica et al., 2005). These events induce a signaling cascade by stimulation of Myeloid Differentiation primary response protein 88 (MyD88) leading to activation and nuclear translocation of transcription factors, such as the Nuclear transcription Factor NF-κB and activation of the innate host defense such as the production of pro-inflammatory cytokines.

CLR/CTL are a family of membrane-bound calcium-dependent receptors that recognize carbohydrate-rich molecules. Among the CLR family, one of the most well-known receptors is the Mannose Receptor (MR), which recognizes mannose molecules/glycolipids present on *Mtb*’s surface such as LAM, ManLAM, and PIM. Stimulation through MR induces production of anti-inflammatory cytokines and fails to activate oxidative responses (Nigou et al., 2001). Previous studies have shown that phagocytosis of mannosylated beads and/or MR-ManLAM interferes with phagosome maturation, highlighting the potential role of glycolipids in the intracellular survival of mycobacteria (Astarie-Dequeker et al., 1999; Kang et al., 2005). A recent analysis of SNPs in the *MRC1* gene within a Chinese population has suggested a possible association of selected SNPs and the susceptibility of individuals to pulmonary TB (Zhang et al., 2012). Moreover, Mincle, Dectin-1 and -2, and Dendritic Cell immunoActivating Receptor (DCAR) also belong to the CLR sub-family and represent probably the most well-known CLR expressed on macrophages. The Mincle receptor specifically recognizes mycobacterial cord factor Trehalose-6,6-dimycolate (TDM), which likely represents the most abundant glycolipid in the mycobacterial cell wall (Ishikawa et al., 2009). Ligation of TDM to Mincle induces several responses such as production of pro-inflammatory cytokines, generation of Th1/Th17 immune responses and induction of granuloma-genesis (Ishikawa et al., 2009; Mishra et al., 2017). It is known that Dectin-1 recognizes β-glucans in fungal pathogens but the precise *Mtb*’s PAMP is not known (Dinadayala et al., 2004). It has been showed that Dectin-1 is important for the innate immunity recognition of *Mtb* and for inducing Th1 and Th17 responses, independently of TLR2 recognition (van de Veerdonk et al., 2010). Dectin-2 has been recently shown to induce host immune responses against *Mtb* infection through the recognition of ManLAM (Yonekawa et al., 2014). DCAR recognizes PIM to induce Th1 responses during *Mtb* infection (Toyonaga et al., 2016). One other particular CRL/CTL, named DCSIGN/CD209, expressed by dendritic cells and macrophages recognizes conserved sugar motifs in a number of viruses, parasites, and bacteria, including *Mtb* (Tailleux et al., 2003; Tanne and Neyrolles, 2010).

Fc receptors and Complement Receptor (CR) are strongly expressed on surface of macrophages. CR3 plays a key role in the phagocytosis of *Mtb* by macrophages with recognition of *Mtb* polysaccharides or PIM (Villeneuve et al., 2005).

Scavenger Receptors are expressed on the cell surface of mammalian monocytes and macrophages and recognized oxidized or acetylated lipoproteins. During *Mtb* infections, the Macrophage Receptor with Collagenous (MARCO) structure is the most studied. MARCO recognizes TDM and cooperates with TLR2 to induce the activation of the transcriptional factor NF-κB and secretion of pro-inflammatory cytokines (Bowdish et al., 2009).

**FIGURE 1:**
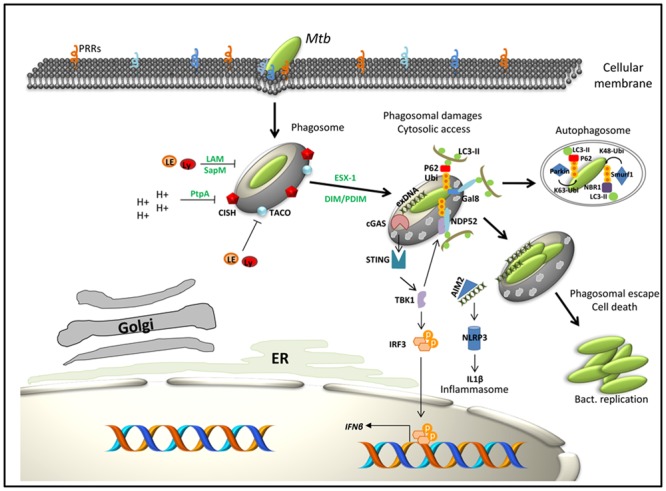
Working model for selected components intervening in the immune subversion strategies of *Mtb* as well as macrophage factors that contribute to host defense. In green: bacterial factors; in black: macrophage factors. Interaction of *Mtb* with macrophage PRRs triggers bacteria internalization. The first strategy of *Mtb* to conquer macrophages is to inhibit phagosome maturation and acidification through the expression or secretion of bacterial factors (LAM, SapM, PtpA) or by subverting host pathways such as TACO or CISH signaling. Bacteria then progressively access to the cytosol through the action of the ESX-1 secretion system and DIM/PDIM. When in contact with the cytosol, bacteria get targeted by ubiquitin ligases (Parkin, Smurf1) and/or activate specific cytosolic recognition pathways (gal8, cGAS, or AIM2). This latter step induces expression of type I IFN, activation of the NRLP3 inflammasome and initiation of autophagy. Lastly, cytosolic *Mtb* induces host cell death programs (apoptosis and necrosis) to enhance its dissemination.

Finally, cytosolic DNA sensors have also been described as PRR, which can recognize the presence of mycobacterial DNA in the cytosol (Manzanillo et al., 2012). This process is dependent on phagosomal rupture induced by ESX-1 and is mediated via the cytosolic sensors cGAS or AIM2 (Collins et al., 2015; Majlessi and Brosch, 2015; Wassermann et al., 2015; Watson et al., 2015; **Figure 1**).

## *Mtb* Strategies to Conquer Macrophages

### Strategy 1: Interference with Phagosome Maturation

Macrophages are acting as the first line of defense against pathogenic invaders. After internalization, pathogens are trapped in a vacuole called phagosome, which immediately undergoes sequential fusion events to acquire microbicidal and degrading characteristics by a process called maturation. The dynamic of phagosome maturation is actively regulated by the network of Rab GTPases, proteins that sequentially drive the phagosome progression from early to later stages of maturation. Rab GTPases (Rab) contribute to the identity of the endosomal organelle (e.g., Rab5, early endosomes; Rab7, late endosome), regulating membrane-fusion events but also the sorting of protein and lipids through the recycling pathway (Gruenberg and van der Goot, 2006; Gutierrez, 2013; Prashar et al., 2017). Thus, all along the usual maturation process, biological changes of the phagosome are characterized by the specific recruitment of Rab GTPase until the final fusion with lysosomes, which carry a set of hydrolytic enzymes that contribute to pathogen clearance. Recently other regulators of the phagosomal maturation, Rab34, Rab20, and the proneurotropin receptor sortilin, have been described as important for control and elimination of intracellular *Mtb* (Kasmapour et al., 2012; Vazquez et al., 2016; Schnettger et al., 2017). Concurrently with the maturation process, the pH of phagosomes quickly drops from neutral to 5, through a high activity of a vesicular proton-pump ATPase (H+ V-ATPase) (Russell et al., 2009). Phagosomal acidification is a prerequisite for intracellular bacterial clearance, as acidic pH is essential for the optimal activity of lysosomal digestive enzymes and for reactive oxygen species production (Vieira et al., 2002; Sun-Wada et al., 2009). All along common trafficking pathways within macrophages, pathogens have to face multiple dangers such as exposure to cytosolic pattern recognition or danger receptors. The cytosolic lectin, Galectin8, notably recognizes damaged pathogen-containing vacuoles (including *Mtb* damaged phagosomes), and promotes their elimination by activating anti-bacterial autophagy (Thurston et al., 2012; **Figure 1**). Additionally, Ubiquitin ligases Parkin, Ubiquilin1, and Smurf1 recognize intracellular *Mtb* and enhance its clearance through an ubiquitin-mediated autophagy pathway (Manzanillo et al., 2013; Sakowski et al., 2015; **Figure 1**).

To survive in this harsh environment, *Mtb* developed a wide range of strategies to counteract macrophages defenses. *Mtb* indeed triggers rapid interferences in phagosome functions by inhibiting the phagolysosome biogenesis. Unless the macrophage is activated by inflammatory cytokines, the mycobacterial vacuole fails to mature along the normal endocytic pathway, and retains features typical of an immature endosome. Just after macrophage uptake, mycobacterial phagosome transitory recruits early endosomal markers Rab5 and remains accessible to the marker of recycling endosome Rab11 (Via et al., 1997; Tailleux et al., 2003; Vergne et al., 2004). In addition, Coronin1, also called TACO, is reported to be recruited and retained at the phagosomal surface where it activates calcium–calcineurin signaling to block the fusion of lysosomes with mycobacterial phagosomes (Jayachandran et al., 2007; **Figure 1**). Consistent with these immature characteristics, this organelle lacks the late endosomal and lysosomal markers Rab7 and CD63, as well as mature and active forms of various lysosomal hydrolases, including cathepsin D (Clemens and Horwitz, 1995; Via et al., 1997; Ullrich et al., 1999). Both bacterial ManLAM and the secreted phosphatase SapM have been shown to inhibit the activity of membrane trafficking regulatory lipid phosphatidylinositol 3-phosphate (PI3P), impairing the phagosomal acquisition of the lysosomal cargo and the delivery of hydrolytic enzyme from the Golgi network (Fratti et al., 2001, 2003; Vergne et al., 2005; **Figure 1**).

Blockade of phagosomal acidification is also a key feature for the intracellular survival of pathogens. Consistently, *Mtb* has developed at least three different strategies aiming to inhibit H+ V-ATPase complex assembly and its subsequent fusion with the phagosomal membrane in order to stabilize the phagosomal pH between 6.2 and 6.5 (Sturgill-Koszycki et al., 1994). Indeed, the phosphatase PtpA secreted by *Mtb* inhibits the assembly of H+ V-ATPase machinery by direct interaction with the subunits H of this complex (Wong et al., 2011). Additionally, interaction of TDM with C-type lectin receptor Mincle has been shown to delay phagosomal maturation and acidification (Axelrod et al., 2008; Patin et al., 2017). As parallel mechanism, we recently reported that *Mtb* depletes H+ V-ATPase from its phagosome by co-opting the function of a host immune regulator, i.e., cytokine-inducible SH2 containing protein (CISH), which selectively targets the H+ V-ATPase subunit A for ubiquitination and degradation by the proteasome (Queval et al., 2017; **Figure 1**). The control of the pH is decisive not only for the survival of *Mtb* but also for the further processing of the mycobacterial phagosome. Indeed, early blockade of the acidification process is a pre-requisite for the ESX-1 dependent phagosomal rupture and the access of *Mtb* to the cytosol of the macrophage (Simeone et al., 2015).

### Strategy 2: Getting Access to the Cytosol

The intracellular localization of *Mtb* inside host cells has been studied since the 1970s. The seminal work of Armstrong and Hart (1971) showed in mouse peritoneal macrophages that were infected with viable or non-viable *Mtb* and BCG strains that mycobacteria can be observed by electron microscopy (EM) inside phagosomes that have blocked the phagosome–lysosome fusion (Armstrong and Hart, 1971). In following years, further EM studies also observed *Mtb* outside the phagosome under certain conditions (Leake et al., 1984; Myrvik et al., 1984; McDonough et al., 1993), whereas others could not observe mycobacteria in the cytosol by EM (Xu et al., 1994). Some of these disparities were thought to have been caused by differences in the EM conditions and protocols used. More recently, using sophisticated cryo-immunogold EM, van der Wel et al. (2007) have challenged the dogma of the exclusive intracellular localization of *Mtb* and have described the existence of cytosolic *Mtb* in THP-1 human macrophage-like cells at 4 days post-infection whereas the BCG strain did not show such a distribution (van der Wel et al., 2007; Houben et al., 2012). This result has been correlated with the function of the ESX/T7S system in *Mtb*, which is absent from BCG due to the ESX-1 deletion. However, given the situation that the suggested paradigm shift was entirely based on ultrastructural observations generated by electron microscopy, the presence of *Mtb* in the cytosol has remained controversial for some time. The development of a Fluorescent Resonance Energy Transfer (FRET) for detection of mycobacteria that have ruptured the phagosome and have established cytosolic contact has been an important advance to study this delicate and fascinating question (Simeone et al., 2012, 2015). For that purpose, host cells are loaded with a chemical probe that is sensitive to FRET changes based on β-lactamase activity present on the surface of bacteria. The use of this FRET-based technology combined with automated fluorescent microscopy (Simeone et al., 2012) and multicolor quantitative cytofluorometry allowed to explore the role of ESX-1 in the induction of phagosomal rupture and more recently to show that *Mtb* induces phagosomal rupture *in vivo* (Simeone et al., 2015; **Figure 1**).

Based on the results from different groups using independent techniques, it thus became clear that the ESX-1/T7S system plays a primordial role for establishing cytosolic access of selected mycobacteria in host cells (van der Wel et al., 2007; Houben et al., 2012; Simeone et al., 2012, 2015). However, very recently, in independent studies, additional mycobacterial factors have been identified that favor the access of *Mtb* to the cytosol. Indeed, it was found that cytosolic access of *Mtb* only occurs when the production and the export of the outer membrane lipids (DIM/PDIM) are intact (Augenstreich et al., 2017). DIM/PDIM are key virulent lipids and play important roles in host-pathogen interaction. Their presence favors intracellular bacterial replication through arrest of phagosomal acidification by excluding the vacuolar proton-ATPase from the phagosomal membrane (Astarie-Dequeker et al., 2009) and they are also involved in the death of macrophages (Passemar et al., 2014). The use of monoclonal antibodies against Galectin-3 and ubiquitinated proteins for identification of damaged phagosomal-membranes (Wong and Jacobs, 2011), in parallel to a FRET-based cytofluorometric approach for detection of phagosomal rupture, demonstrated the implication of DIM/PDIM in phagosomal rupture (**Figure 1**). This study showed that both the ESX-1 system and a functional DIM/PDIM production were required to cause phagosomal damage and rupture, which ultimately leads to host cell death (Augenstreich et al., 2017). The implication of DIM/PDIM in this phenomenon has independently been confirmed by a study that investigated the DNA interaction and the regulon of a transcriptional repressor (Rv3167c), which was found to control the DIM/PDIM operon and to impact phagosomal escape (Quigley et al., 2017). Additional confirmation came from a third study that carried out a multiparametric analysis, combining pathogen and host phenotypes, and found similar profiles for ESX-1 and DIM/PDIM loss-of-function mutants (Barczak et al., 2017). Phospholipases have been described to play a role in the escape of the bacteria from phagosome to cytosol by acting together with pore-forming proteins such as listeriolysin from *Listeria monocytogenes* (Cossart, 2011). The *Mtb* genome presents four phospholipases PlcA-D, whereby in the reference strain *Mtb* H37Rv PlcD has already been naturally inactivated by an IS*6110*-mediated deletion (Cole et al., 1998). The use of FRET-based cytofluorometry, however, showed that Plcs of *Mtb* do not seem to play a role in the phagosomal rupture as triple/quadruple Plc deletion mutants continued to generate positive signals in the phagosomal rupture assay, similar to wild-type strains (Le Chevalier et al., 2015).

Few studies report data on the implication of host factors involved in the induction of phagosomal rupture. A first result was obtained by the use of the FRET-cytofluorometry approach for the study of *Mtb* infection in macrophages carrying a non-functional *nramp* gene, encoding the Natural Resistance-Associated Macrophage Protein (Nramp-1), a phagosomal bivalent cation transporter implicated in phagosomal acidification and pH regulation (Simeone et al., 2015). This approach showed that initial blockage of the acidification of the phagosome is necessary to allow bacteria to survive and to induce phagosomal rupture (Simeone et al., 2015). Another host element that has been suggested in this context is the cytosolic phospholipase A2 (cPLA2). This enzyme plays a critical role in both phagosomal trafficking and export of cargo from the various endocytic comportments and permeabilizes the endosomal membrane in *Mtb*-infected macrophages (Lee et al., 2011). Treatment of *Mtb*-infected macrophages with an inhibitor of this enzyme induces a marked reduction of cytosolic bacteria as observed by EM (Jamwal et al., 2016).

Cytosolic contact of *Mtb* thus seems to be fundamental in mycobacterial host-pathogen interaction, influencing both the fate of the host cell and the bacteria. Indeed, the recognition of mycobacteria-associated patterns by the cytosolic receptors of the innate immunity determines innate and adaptive immune responses (Gröschel et al., 2016). Following steps exist: (i) DNA is sensed by cGAS, which synthesizes the second messenger cGAMP from ATP and GTP. cGAMP activates the Endoplasmic Reticulum (ER) associates Stimulator of IFN Genes (STING) and downstream TBK-1-IRF-3-IFN-β signaling axis (**Figure 1**). This effect leads to the expression of type I IFNs, such as IFN-α/β, which are thought to be disadvantageous to the host during *Mtb* infection, (ii) the cytosolic *Mtb* DNA may be sensed by AIM-2, which contributes partially to the activation of the NRLP3 inflammasome axis and release of mature IL-1β and IL-18 (Collins et al., 2015; Wassermann et al., 2015; Watson et al., 2015; Kupz et al., 2016), and (iii) the ESX-1-mediated cytosolic translocation of mycobacterial DNA results in the activation of TBK-1 which initiates the recruitment of LC3-II involved in autophagic activity (Romagnoli et al., 2012; Watson et al., 2012; **Figure 1**). The last two points, in contrast to the first one, might be considered as more beneficial for the host. Thus, during infection, different and sometimes opposite responses govern the balance between the benefit for the mycobacteria and for the host. Nonetheless, during the infectious process mycobacteria are not necessarily constrained within the host cells, and may escape from the microbicidal environment of macrophages by disseminating inside the organism. It has been notably reported in the Zebra fish model that *Mycobacterium marinum* membrane Phenolic Glycolipids (PGL) trigger a STING-dependent secretion of monocyte chemoattractant protein 1 (MCP1; also called CCL2) by infected-resident macrophages, resulting in the recruitment of circulating monocytes and a subsequent transfer of the bacteria from tissue resident- to circulating macrophages (Cambier et al., 2017).

Finally, in the context of vaccination, the induction of ESX-1-mediated cytosolic responses seems to be beneficial for increased protective efficacy provided by recombinant BCG strains over parental BCG strains (Kupz et al., 2016; Groschel et al., 2017).

### Strategy 3: The Control of Host Cell Death

The control of host cell death allows *Mtb* to escape host defenses and to take the power on the pathogenesis control. For decades, host cell death upon *Mtb*-infection has been controversial and apoptosis cell death was considered as the only programmed cell death. Two main types of cell death are known for elimination of infected cells: (i) apoptosis or programmed cell death and (ii) necrosis.

(i) From a morphological point of view, apoptosis is defined by plasma membrane bleeding, cell body shrinkage, nuclear condensation and fragmentation, and formation of apoptotic bodies, which are membrane-bound cell fragments rapidly phagocytosed by neighboring cells and resident phagocytes (Lamkanfi and Dixit, 2010). From a biochemical point of view, apoptosis induces a decrease in mitochondrial inner transmembrane potential, activation of selective proteases, cleavage of chromosomal DNA, and various cellular proteins and translocation of phosphatidylserine from the inner to the outer plasma membrane (Behar et al., 2011). Apoptosis does occur via TNF-α activation and caspase 3 and 8 activation. Suppression of inflammation allows to limit tissue damage. Apoptosis of infected cells is considered as a benefit for the host. Indeed, it allows elimination of a favorable environment for replication of the pathogen, and provides an important source of bacterial antigens that can stimulate *Mtb*-specific T-cell immunity (Behar et al., 2011). Apoptosis has been directly linked to an increase CD8+ T-cell response via cross-presentation and enhances class II MHC-restricted antigen presentation (Behar et al., 2011).
Some *Mtb* genes have been reported to play a role in the inhibition or induction of host cell death, as for example *sodA*, encoding superoxide dismutase A, or *nuoG*, encoding the NADH dehydrogenase 1 subunit G (Velmurugan et al., 2007; Gengenbacher et al., 2016). An *Mtb* gene well known for inducing host cell death is *esxA*, encoding EsxA, which has been described as a pro-apoptotic (Aguilo et al., 2013). The exact role of EsxA in this process remains unclear, but most probably it is the contribution to the access of *Mtb* to the host cytosol, which plays a main role. However, while the biological role of the ESX-1 system in the process remains fully valid, the function of recombinant EsxA as a putative membranolytic molecule was recently questioned, as the lytic activity of EsxA preparations expressed and purified from *Escherichia coli* lysates on red blood cells continued even after digestion with proteinase K, suggesting that some of the previously described pore-forming activity might be simply caused by a selected detergent used during the protein purification process (Conrad et al., 2017). Further studies are needed to clarify the role of EsxA in the process.
(ii) In contrast to apoptosis, necrosis is characterized by loss of plasma membrane integrity, cytoplasmic organelles swelling such as mitochondria and cell nuclei, release of cytoplasmic and nuclear contents to the extracellular space, hydrolysis of chromatin and DNA and is caspase-independent cell death (Lamkanfi and Dixit, 2010). It was previously thought that virulent *Mtb* inhibits apoptosis and triggers necrosis to evade innate immunity and thus to delay the initiation of adaptive immunity. On the contrary, attenuated *Mtb* induces macrophage apoptosis, which reduces bacterial viability (Behar et al., 2010). However, from different recent studies, it can be hypothesized that apoptosis induced by virulent *Mtb* favors dissemination of bacilli (Aguilo et al., 2013; Augenstreich et al., 2017), while necrosis tends to enhance bacterial replication (Dallenga et al., 2017; Lerner et al., 2017).

Colonization of macrophages by *Mtb* is a highly disputed process that depends on the ability of the pathogen to escape from macrophage killing and the capacity of the macrophages to control the bacterial proliferation.

## Perspectives

It is clear that the interaction of *Mtb* with the macrophage has major impact on the outcome of infection, whereby both mycobacterial and host factors play important roles. The accumulated vast knowledge in recent years on this process might also turn out as important for developing potential new strategies in the fight against TB, concerning vaccines and host-directed therapies. For example, BCG complemented with the functional ESX-1 system of *Mtb* showed better efficacy to protect against disseminated TB in mice and guinea pigs (Pym et al., 2003; Kupz et al., 2016). However, this recombinant strain named BCG::RD1 has also been shown to be more virulent than the wild-type BCG strain (Pym et al., 2002). As one possibility to reduce this enhanced virulence, introduction of selected mutations in the *esxA* gene of the cloned ESX-1 locus have shown some effect (Bottai et al., 2015). Alternatively, the use of an ESX-1 secretion system taken from a less virulent mycobacterium than *Mtb* for the complementation of BCG seems also promising. Indeed, recombinant BCG expressing the ESX-1 system from *Mycobacterium marinum*, BCG::ESX-1^Mmar^, has been recently shown to be low virulent and more protective than parental BCG strains in different murine models of infection (Groschel et al., 2017). This strain enhances NLRP3 inflammasome activation and induces type I IFN production and stronger CD8^+^ and CD4^+^ T-cell responses (Groschel et al., 2017).

Using attenuated live *Mtb* strains as vaccines is another alternative approach, presenting the advantage that these strains naturally carry genetic regions encoding for important immunodominant antigens that might be absent from BCG in combination with sufficient safety, provided by the introduction of deletions in virulence genes. The live-attenuated MTBVAC is a good example (Aguilo et al., 2017). MTBVAC attenuation is based on two independent stable genetic deletions, without antibiotic resistance markers, introduced into *phoP* and *fadD26*, affecting the production and secretion of selected lipidic and proteic virulence factors of *Mtb*. MTBVAC presents promising features in preclinical animal models and is presently being evaluated in a phase I clinical trial in newborns in South Africa (Marinova et al., 2017). Other attenuated *Mtb* strain, presently in preclinical development with promising results in murine infection models, is the *Mtb*Δppe25-pe19 strain, in which selected PE and PPE proteins of the ESX-5 secretion system have been deleted, but which retains an intact ESX-1 secretion system (Bottai et al., 2012; Sayes et al., 2012, 2016).

Concerning drug treatment of TB, most of the drugs used today date from the 1950s to 1960s, with a few exceptions, such as bedaquiline, a recently discovered ATP synthase inhibitor (Andries et al., 2005) that is currently used successfully in drug regimens against MDR-TB (Diacon et al., 2009). However, additional identification of new active anti-TB drugs is urgently needed. In this perspective, it should be mentioned that remarkable progress has been made in developing new screening approaches that can simultaneously evaluate the anti-bacterial potency and the non-cytotoxic properties of small molecule inhibitors in the intracellular environment of *Mtb*-infected macrophages (Christophe et al., 2009; Pethe et al., 2013; VanderVen et al., 2015). Moreover, targeting of mycobacterial virulence factors might also be a possibility to find alternative treatment approaches (Chen et al., 2010; Bottai et al., 2014). As one example, screening of a compound library recently allowed molecules to be identified that target the ESX-1 secretion system of *Mtb* (Rybniker et al., 2014) and/or the global two-component regulator PhoP/R which among many other virulence factors also regulates ESX-1-mediated secretion (Johnson et al., 2015).

Host-directed therapy (HDT) to treat TB is a relatively new and promising concept that starts to arouse a great interest from the scientific community. Instead of targeting *Mtb* compounds directly, HDT targets the host response, such as the modulation of host inflammatory pathways to reduce inflammation and lungs tissue damages, augmenting cellular anti-microbial mechanisms. In the case of TB-HIV co-infection, HDT may reduce the risk of interaction with antiretroviral drugs. In the case of MDR or XDR-TB, HDT could be added to anti-TB treatment to increase the capacity of the host system to eliminate mycobacteria or to limit tissue damage due to the infection (Wallis and Hafner, 2015; Zumla et al., 2015). For instance, in association with anti-TB drugs, statins, a family of inhibitors of HMG-CoA reductase, originally used to lower cholesterol levels in patients, drastically enhance the efficacy of first-line TB treatments in macrophages and *in vivo* models (Lobato et al., 2014; Parihar et al., 2014; Skerry et al., 2014). Moreover, Vitamin D treatment has been suggested to promote the expression anti-microbial peptide Cathelicidin by macrophages, and thus lowering of the intracellular survival of *Mtb* (Liu et al., 2006; Wheelwright et al., 2014).

Some HDTs are in clinical human trials or preclinical animal studies, such as anti-inflammatory therapies, modulation of inflammation by phosphodiesterase inhibitors, eicosanoid modulation, non-steroidal anti-inflammatory drugs, high-dose vitamin D application, alteration of lipid metabolism, as well as new HDT that targets autophagy (Tobin, 2015). Autophagy appears to be a promising pathway to target for the development of drugs. Interestingly, anti-mycobacterial activities of both statins and Cathelicidin have been correlated with their ability to enhance the autophagy pathway (Yuk et al., 2009; Hoyer-Hansen et al., 2010; Parihar et al., 2014). As such, HDT opens new avenues for individualized TB therapies.

## Conclusion

We have discussed a selection of amazing strategies of *Mtb* and host cells in their battle about survival and death in host-pathogen interaction that remains a highly interesting research domain. Driven by an advancing technical progress, many new insights were obtained in recent years and have often led to changes in long-lasting hypotheses and theories, a finding which gives hope that in the upcoming years more progress will further contribute to the knowledge how to better fight *Mtb* and reduce the burden of TB in the world.

## Author Contributions

All authors listed have made a substantial, direct and intellectual contribution to the work, and approved it for publication.

## Conflict of Interest Statement

The authors declare that the research was conducted in the absence of any commercial or financial relationships that could be construed as a potential conflict of interest.
